# Therapeutic applications of miRNA in the management of obesity and osteoporosis

**DOI:** 10.1007/s40200-025-01589-6

**Published:** 2025-03-03

**Authors:** Sakhavat Abolhasani, Yasin Ahmadi, Yavar Rostami, Mostafa Bafandeh Zendeh, Davood Fattahi

**Affiliations:** 1 Department of Basic Sciences and Health, Sarab Faculty of Medical Sciences, Sarab, East Azerbaijan Iran; 2 Department of Medical Laboratory Science, Komar University of Sciences and Technology, Sulaymaniyah, Kurdistan Region Iraq; 3 School of Pharmacy and Biomolecular Sciences, Liverpool John Moores University, Liverpool, UK

**Keywords:** Adipogenesis, Bone Metabolism, MicroRNAs, Molecular Therapy, BMSCs Differentiation, Obesity, Osteogenesis, Osteoporosis

## Abstract

Obesity and osteoporosis are interrelated global health challenges, both characterized by dysregulated bone metabolism and adipose tissue dynamics, contributing to increased fracture risk and systemic complications. Emerging evidence underscores the pivotal role of microRNAs (miRNAs) as regulatory molecules governing the intricate balance between adipogenesis and osteogenesis, thereby providing a molecular link between these two conditions. Both disorders are characterized by intricate alterations in bone metabolism and adipose tissue dynamics, which increase the risk of fractures and systemic complications. Recent advancements in molecular biology have identified miRNAs as crucial regulators of these disorders, influencing the differentiation of bone marrow mesenchymal stem cells (BMSCs) into osteoblasts (bone-forming cells) and adipocytes (fat-storing cells). This review provides a comprehensive analysis of the dual role of miRNAs in modulating osteogenesis and adipogenesis, with a particular focus on their implications in disease progression and therapeutic strategies. It first explores how specific miRNAs regulate critical energy metabolism, inflammation, and bone remodeling pathways. By integrating insights from molecular biology, endocrinology, and clinical practice, the review highlights the therapeutic potential of miRNA-based interventions. Targeting specific miRNAs could restore the balance between adipogenesis and osteogenesis, offering innovative approaches to simultaneously address obesity and osteoporosis. These proposed strategies hold promise for improving patient outcomes by mitigating fracture risk, enhancing bone density, and addressing metabolic dysfunctions associated with obesity. Ultimately, future research should focus on translating these molecular insights into clinical applications to develop effective therapies that tackle the complex interplay between these prevalent conditions.

## Introduction

Obesity and osteoporosis are two prevalent global health issues significantly affecting morbidity and mortality rates, creating a substantial burden on healthcare systems worldwide [1]. Both conditions are characterized by notable alterations in bone metabolism and adipose tissue dynamics, which together lead to an increased risk of fractures and a range of systemic complications [2]. Recent research has accentuated the critical role of microRNAs (miRNAs) as key regulators in the pathogenesis of these diseases, suggesting their significance as valuable biomarkers and therapeutic targets [3].

miRNAs are small, non-coding RNA molecules playing a fundamental role in the post-transcriptional regulation of gene expression [4]. They are involved in various biological processes, including the differentiation of bone marrow mesenchymal stem cells (BMSCs) into osteoblasts, the cells responsible for bone formation, and adipocytes, the cells that store fat [5]. The interplay between obesity and osteoporosis is particularly concerning, as the accumulation of visceral fat can significantly exacerbate the progression of osteoporosis through the promotion of adipogenesis, the process by which pre-adipocytes develop into mature adipocytes, at the expense of osteogenesis, the formation of new bone tissue [6]. Such a shift impairs bone mineralization and alters the bone microenvironment, rendering it less conducive to regeneration and repair [7].

The relationship between obesity and osteoporosis is further complicated by the inflammatory environment associated with excess adipose tissue [8]. Adipose tissue, far from being a passive fat storage depot, functions as an active endocrine organ that secretes pro-inflammatory cytokines and adipokines [9] which negatively affect bone health by promoting osteoclastogenesis, thereby increasing bone resorption and decreasing bone density [10]. Therefore, the dual burden of obesity and osteoporosis can create a vicious cycle, wherein the presence of one condition exacerbates the other, leading to a heightened risk of fractures and other skeletal-related events [11].

Recent studies have shown that specific miRNAs are upregulated in conditions of obesity and osteoporosis, suggesting their potential as biomarkers for disease progression and therapeutic targets [12]. Certain miRNAs regulate pathways that influence both adipocyte differentiation and osteoblast function, thereby establishing a molecular connection between these two conditions [13].

This narrative review aims to consolidate knowledge on miRNA-based therapies for obesity and osteoporosis. The role of miRNAs in regulating the differentiation of BMSCs and their involvement in bone remodeling will be examined. The complex networks of miRNA interactions hold the potential for targeted interventions addressing the interconnected nature of these disorders, ultimately enhancing patient outcomes. As research progresses, further investigation into the roles of miRNAs in these conditions is crucial, as they offer significant potential to improve both the understanding and treatment of obesity and osteoporosis on a global scale. By integrating insights from molecular biology, endocrinology, and clinical practice, comprehensive strategies can be developed to address both the symptoms and underlying mechanisms of these diseases, thereby enhancing the quality of life for affected individuals.

## The role of miRNAs in obesity

Obesity is a complex condition characterized by an excessive buildup of adipose tissue, which increases the risk of various metabolic disorders, such as type 2 diabetes and cardiovascular disease [14]. Recent research has illuminated the critical role of miRNAs in regulating essential biological processes linked to obesity, particularly in the areas of adipogenesis, energy balance, and insulin sensitivity (Table 1) [15].

**Table 1:** miRNA role in obesity: mechanism of action therapeutic potential

miRNA	Role in Obesity	Mechanism of Action	Therapeutic Potential
miR-27a	Promotes the differentiation of preadipocytes into mature adipocytes	Downregulates lipid metabolism genes, enhancing fat storage	Targeting miR-27a may reverse obesity-related metabolic issues [18]
miR-143/145	Regulates fatty acid oxidation and glucose uptake in adipocytes	Modulates pathways critical for energy balance and insulin sensitivity	Potential biomarkers for metabolic health [16]
miR-155	Contributes to inflammation and insulin resistance	Influences metabolic pathways, promoting chronic inflammation	Targeting miR-155 may alleviate insulin resistance [17]
miR-221	Modulates fat metabolism through leptin and TNF-α	Upregulated in obesity; affects adipose tissue metabolic processes	May serve as a target for therapeutic interventions [19]
miR-26b	Associated with brown adipogenesis and energy expenditure	Targets white fat gene expression, promoting energy dissipation	Could enhance brown fat content to combat obesity [20]
miR-34a	Regulates metabolic pathways and apoptosis	Modulates genes linked to energy metabolism and inflammation	May be targeted to improve insulin sensitivity and reduce obesity [21]
miR-122	Involved in lipid metabolism and liver function	Regulates hepatic lipid metabolism and cholesterol balance	Potential to target for treating liver-related obesity conditions [22]
miR-193b	Regulates ectopic fat deposition and glucose metabolism	Modulates pathways affecting insulin sensitivity and adipogenesis	May offer new strategies for managing body fat distribution [23]
miR-126	Involved in endothelial function and inflammation	Regulates angiogenesis, potentially influencing vascular dynamics in adipose tissue	Exploring its role in promoting metabolic health in obesity [24]
miR-708	Linked to lipid accumulation and obesity-induced inflammation	Targets pathways related to lipid metabolism and inflammation	Could be a therapeutic target for obesity-related metabolic disorders [25]

### miRNAs regulating energy metabolism

**miR-143/145** serve as vital regulators of fatty acid oxidation and glucose uptake in adipocytes, impacting metabolic pathways crucial for maintaining energy balance and insulin sensitivity [16]. **miR-155** is implicated in inflammatory responses and the development of insulin resistance, exacerbating the chronic inflammation often observed in obesity, thus impairing metabolic function [17]. **miR-221** influences fat metabolism by modulating the action of leptin and tumor necrosis factor-alpha (TNF-α). Its upregulation in obesity aligns with its capacity to affect metabolic processes linked to the functionality of adipose tissue. **miR-26b,** which is involved in regulating brown adipogenesis and energy dissipation, targets genes involved in white adipose tissue development, thereby fostering increased energy expenditure, which is crucial for combating obesity. **miR-34a** is involved in the regulation of pivotal metabolic pathways and apoptosis. Table 1 provides further information on the impact of miRNAs on energy metabolism.

## The role of miRNA in bone turnover

miRNAs have emerged as crucial regulators of bone homeostasis, influencing the dynamic equilibrium between bone formation and resorption. These small non-coding RNAs modulate gene expression post-transcriptionally, affecting key cellular processes such as osteoclastogenesis, osteoclastogenesis, and mesenchymal stem cell differentiation. Dysregulation of miRNAs has been implicated in various bone disorders, including osteoporosis, were imbalances in bone remodeling led to reduced bone mass and structural fragility. Understanding the role of miRNAs in bone turnover provides insights into their potential as therapeutic targets for bone-related diseases. This section explores the intricate functions of miRNAs in regulating the balance between adipogenesis and osteogenesis, as well as their specific involvement in osteoporosis pathogenesis.

### miRNAs regulating the delicate balance of adipogenesis and osteogenesis

The balance between adipogenesis and osteogenesis is critical for maintaining skeletal integrity and overall health. In the context of obesity, there is a marked upregulation of signaling pathways that favor the differentiation of mesenchymal stem cells (MSCs) into adipocytes rather than osteoblasts. This shift not only reduces the rate of new bone formation but also compromises the structural quality of existing bone, making it more susceptible to fractures and other skeletal lesions [26, 27].

miRNAs play a pivotal regulatory role in this context. Specific miRNAs, such as miR-27a, are instrumental in promoting the differentiation of bone marrow BMSCs into adipocytes by targeting genes involved in lipid metabolism [28]. Conversely, other miRNAs, such as miR-21, are known to enhance osteogenic differentiation and promote bone formation by modulating key signaling pathways associated with osteoclastogenesis [29]. Regarding their significant role in bone turnover, targeting miR-27a and miR-21 holds significant potential for improving bone health by regulating key processes such as osteogenesis and osteoclastogenesis. Modulating specific miRNAs could enhance bone formation, reduce resorption, and restore balance in bone remodeling, contribution a promising approach for treating osteoporosis [30].

Inhibition of miR-27a holds significant potential for the treatment of osteoporosis, given its key role in promoting adipogenesis and inhibiting osteogenesis. By modulating lipid-related pathways, strategies to suppress miR-27a could mitigate excessive fat accumulation while simultaneously promoting osteogenic differentiation [30]. Restoring the balance between adipogenesis and osteogenesis through miR-27a inhibition may enhance both metabolic function and skeletal health, offering a promising therapeutic approach for osteoporosis management [31].

Given its critical role in enhancing osteogenic differentiation of MSCs through modulation of key signaling pathways, including the SMAD7-SMAD1/5/8-RUNX2 axis, miR-21 promotes bone formation and increases bone density [32, 33]. Additionally, it addresses metabolic dysfunctions often associated with obesity. Targeting miR-21 could reduce fracture risk while simultaneously regulating metabolic processes, making it a promising candidate for therapeutic interventions [34]. Its dual functionality in both bone formation and metabolic regulation highlights its therapeutic potential for treating osteoporosis and related metabolic disorders.

### The role of miRNAs in osteoporosis

Osteoporosis is a systemic skeletal condition characterized by a pronounced decline in bone mass and an elevated risk of fractures. This disorder results from an imbalance in bone remodeling, wherein the resorption of bone by osteoclasts exceeds the formation of bone by osteoblasts [35]. Recent studies have highlighted the pivotal role of miRNAs as essential regulators of bone metabolism, influencing the differentiation and functional activity of both osteoblasts and osteoclasts [36].

In the pathology of osteoporosis, several specific miRNAs have been extensively studied and identified as key contributors to bone metabolism.

**miR-21** is recognized for its significant role in promoting osteogenic differentiation in MSCs. miR-21 modulates critical signaling pathways, particularly the SMAD7-SMAD1/5/8-RUNX2 axis, which is essential for the maturation and functional competence of osteoblasts. By enhancing the expression of key osteogenic factors, miR-21 not only facilitates bone formation and mineralization but also plays a protective role against the bone loss commonly associated with osteoporosis [37].

**miR-34a is** rrenowned for its inhibitory effects on osteoclastogenesis, and it is integral in mitigating bone resorption. This miRNA impacts target genes that govern the differentiation and activity of osteoclasts, thereby contributing to the maintenance of bone density and overall structural integrity [38]. Furthermore, miR-34a’s influence on inflammatory pathways linked to obesity suggests that targeting this miRNA may simultaneously address chronic inflammation and metabolic dysfunction, providing a comprehensive strategy for managing both osteoporosis and related metabolic conditions.

**miR-702-5p** has been implicated in diabetic osteoporosis, acting to regulate both the proliferation and mineralization of osteoblasts via the OGN/Runx2 signaling pathway. Dysregulation of miR-702-5p may lead to impaired bone formation observed in diabetic patients, indicating that restoring its normal activity could provide new therapeutic strategies for addressing osteoporosis in this demographic [39]. Table 2 provides further information and examples of the miRNAs involved in the regulation of bone turnover.

**Table 2:** miRNAs in osteoporosis: mechanism of action and therapeutic potential

miRNA	Role in Osteoporosis	Mechanism of Action	Therapeutic Potential
miR-21	Promotes osteogenic differentiation in MSCs	Modulates the SMAD7-SMAD1/5/8-RUNX2 signaling pathway, promoting osteoblast maturation and function	Targeting miR-21 may help prevent osteoporosis-related bone loss [37]
miR-34a	Inhibits the formation of osteoclasts, reducing bone resorption	Targets genes involved in osteoclast differentiation and activity, helping maintain bone density	A promising therapeutic target for osteoporosis management [38]
miR-702-5p	Regulates osteoblast proliferation and mineralization in diabetic osteoporosis	Influences the OGN/Runx2 signaling pathway; dysregulation is related to poor bone formation	Restoring miR-702-5p function may assist in diabetic osteoporosis treatment [39]
miR-146a	Involved in inflammatory responses affecting bone metabolism	Regulates osteoclast activity and inflammatory cytokine production, impacting bone remodeling	Potential target for managing inflammation-related bone loss [40]
miR-133a	Modulates osteoblast differentiation and function	Affects key transcription factors involved in osteogenesis	Targeting miR-133a could enhance bone formation [41]
miR-29	Regulates bone extracellular matrix composition	Suppresses matrix metalloproteinases (MMPs), influencing remodeling	Targeting miR-29 could improve bone quality [42]
miR-129	Enhances osteogenic differentiation in stem/progenitor cells	Regulates genes associated with osteoblast differentiation	Potential role in enhancing bone regeneration [43]
miR-503	Inhibits osteoclast genesis and promotes osteoblast function	Modulates RANKL and OPG expressions, balancing cell activity	Could be a therapeutic target to prevent bone loss [44]
miR-219	Crucial for osteoblast differentiation and bone formation	Targets inhibitors of osteogenesis to facilitate osteoblast maturation	improve therapeutic strategies for osteoporosis [45]
miR-146b	Regulates osteoclast differentiation and bone resorption	Involves the NF-κB pathway, affecting inflammation and metabolism	Potential target for treating inflammation-related osteoporosis [46]
miR-185	Involved in bone remodeling and development	Modulates genes related to both osteoclasts and osteoblasts	May provide insights into new therapeutic strategies in osteoporosis [47]

SMAD: Suppressor of Mothers Against Decapentaplegic. RANKL: Receptor Activator of Nuclear factor Kappa-β Ligand. OPG: Osteoprotegerin. MMP: Matrix Metalloproteinases. NF-κB: Nuclear Factor Kappa Light Chain Enhancer of Activated B Cells. OGN: Osteoglycin.

#### Therapeutic potential of targeting miRNAs in osteoporosis

Research has identified several potential therapeutic impacts of various miRNAs for managing osteoporosis. miR-19a-3p has been demonstrated to promote osteogenic differentiation, potentially decelerating the progression of osteoporosis [48]. By inhibiting histone deacetylase 4 (HDAC4), miR-19a-3p enhances the expression of osteogenic markers, facilitating the transition of MSCs into osteoblasts and thereby contributing to bone formation [49]. miR-221 is recognized for its role in suppressing osteoclastogenesis, offering a strategic approach to improve bone density by reducing the differentiation and activity of osteoclasts, which are responsible for bone resorption [50]. Targeting miR-221 could therefore help establish a favorable balance between bone formation and resorption, addressing a fundamental concern in osteoporosis [51]. Emerging evidence suggests that miR-132 may regulate osteoblast activity and bone formation [52]. By targeting genes within the Wnt signaling pathway, miR-132 promotes osteogenic differentiation, presenting it as a viable candidate for therapeutic intervention in osteoporosis [52].

miR-146a known for its involvement in inflammatory processes, modulates osteoclast activity and cytokine production [40]. By influencing these mechanisms, miR-146a may serve as a therapeutic target for managing inflammation-related bone loss, particularly in cases of postmenopausal osteoporosis [53]. miR-29b is associated with the regulation of extracellular matrix components and bone remodeling. By downregulating matrix Metalloproteinases (MMPs), miR-29b may improve bone quality and density, representing a novel target for osteoporosis treatment [54].

The identification and characterization of these miRNAs not only enhance our understanding of the molecular foundations of osteoporosis but also underscore their potential as innovative therapeutic agents [12]. By selectively targeting miRNAs involved in bone remodeling, it may be possible to devise novel treatment strategies that effectively address both the prevention and management of osteoporosis [55]. As research further elucidates the complex roles of miRNAs in bone metabolism, their potential application in clinical settings as biomarkers and therapeutic targets will likely improve patient care and outcomes in osteoporosis treatment [56].

## Combining therapeutic insights from obesity and osteoporosis

The intricate interrelationship between obesity and osteoporosis offers a unique opportunity for the development of integrative therapeutic strategies. Both conditions share common underlying molecular mechanisms, particularly those involving the regulation of bone metabolism and adipose tissue dynamics. Recent research has demonstrated that the accumulation of visceral adipose tissue not only exacerbates the progression of osteoporosis but also significantly influences the systemic metabolic environment. This creates a detrimental cycle where each condition exacerbates the other, negatively affecting overall health [57].

### Dual-targeting approach

The formulation of dual-targeting therapeutic strategies centered on miRNA modulation presents a compelling approach to tackle the interrelated pathways of obesity and osteoporosis. By focusing on miRNAs that concurrently regulate adipogenic and osteogenic processes, practitioners can devise more effective treatment regimens that directly address the underlying etiological factors of these conditions. For instance, a therapeutic strategy that targets miR-27a to inhibit fat accumulation while simultaneously promoting bone formation through the enhancement of miR-21 could yield synergistic effects, significantly advancing both metabolic and skeletal health [30, 58].

Moreover, the benefits of such dual-targeting strategies could extend beyond mere enhancements in bone density and reductions in fracture risk. By simultaneously addressing issues such as insulin resistance, chronic inflammation, and other metabolic dysfunctions related to obesity, miRNA-based therapies could offer a complete model of patient care. This integrated approach has the potential to disrupt the vicious cycle often observed between obesity and osteoporosis, ultimately developing improved clinical outcomes and significantly enhancing the quality of life for individuals affected by these interconnected conditions [27, 57]. By aligning therapeutic strategies with the biological mechanisms involved, researchers and clinicians can develop comprehensive treatments that address both symptoms and root causes of obesity and osteoporosis.

## Horizon in clinical applications of miRNAs as biomarkers for treatment monitoring

**Fig. 1:**
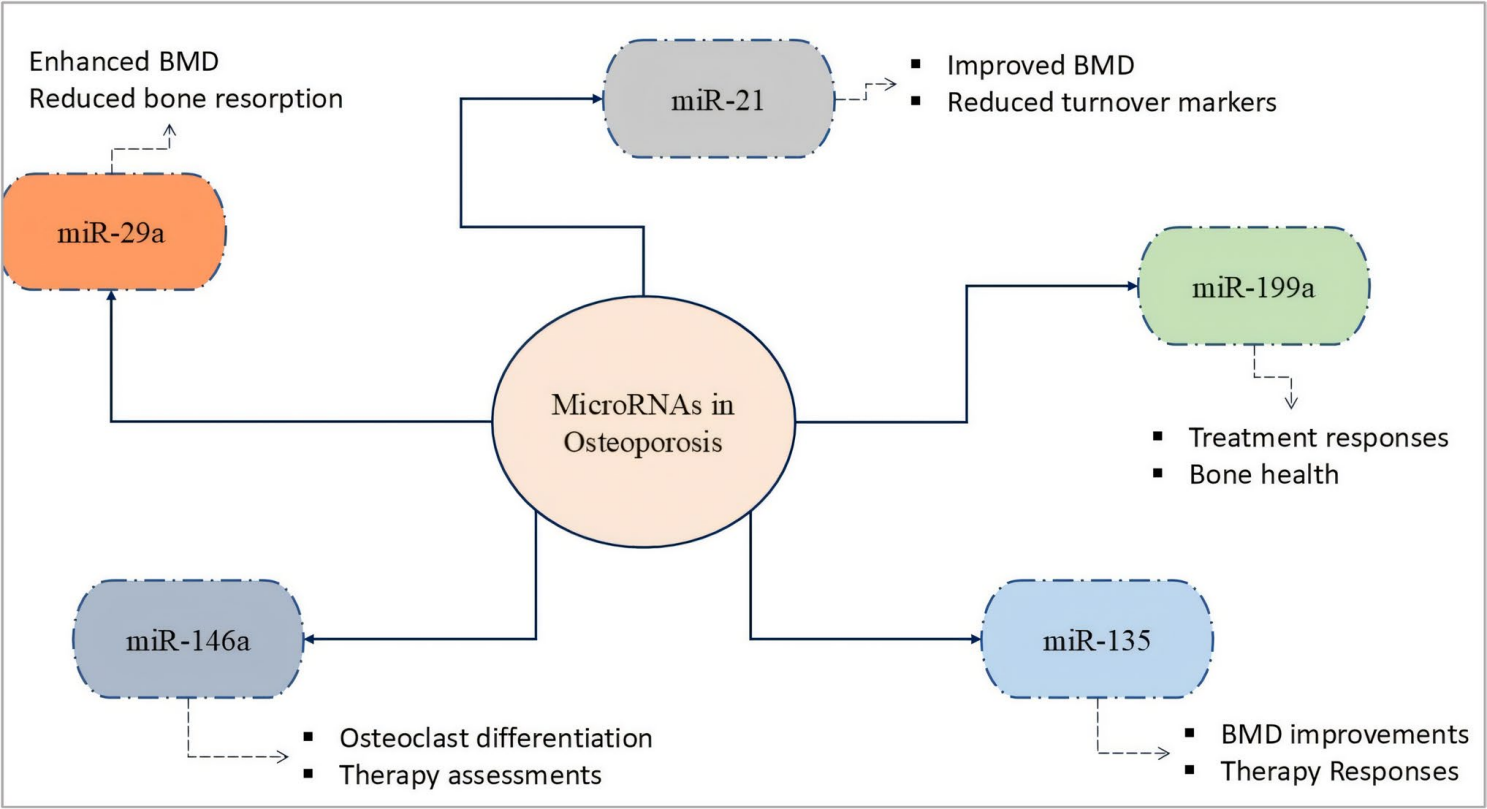
The roles of specific miRNAs (miR-21, miR-29a, miR-135, miR-146a, and miR-199a [60–63] as biomarkers in osteoporosis. These miRNAs play crucial roles in regulating bone health, influencing factors such as bone mineral density (BMD), bone turnover markers (BTMs), treatment responses, and osteoclast differentiation

Recent research has investigated the potential of circulating miRNAs as biomarkers for assessing therapeutic responses in patients with osteoporosis, particularly those receiving treatments such as denosumab [59]. Specific changes in miRNA levels have been linked to improvements in bone mineral density (BMD) and variations in bone turnover markers (BTMs), indicating their practical application in clinical settings for evaluating treatment effectiveness [60–63]. A longitudinal study revealed that after two years of denosumab therapy, several miRNAs showed significant alterations that corresponded with enhanced BMD and reduced BTMs [64] (Fig. 1).

This finding underscores the promising role of these molecules in monitoring patient progress and responses to treatment. Thus, further exploration of the mechanistic pathways by which miRNAs influence bone metabolism is necessary to validate their reliability as indicators of therapeutic outcomes [65].

## Challenges and future directions

Despite the considerable promise that miRNAs hold for the treatment of conditions such as obesity and osteoporosis, a range of significant challenges must be addressed to improve their clinical applicability [66]. One of the foremost obstacles in the deployment of miRNA therapies is the effective delivery of these molecules to their intended target tissues [67]. The intrinsic properties of RNA oligonucleotides create substantial barriers; they are prone to degradation by nucleases, exhibit low permeability across cellular membranes, and are swiftly eliminated from circulation through renal processes [68]. Collectively, these factors complicate the successful targeting and delivery of miRNAs to the requisite sites of action. As a result, there is a pressing need for innovative delivery mechanisms that enhance the stability and bioavailability of miRNA therapeutics in vivo [69].

In addition to delivery issues, ensuring the specificity of miRNA-based interventions remains a critical challenge. Each miRNA has the potential to interact with numerous mRNA targets, which increases the risk of off-target effects. These unintended interactions can lead to adverse outcomes and toxicity, thereby complicating the therapeutic framework surrounding miRNA applications [70]. Addressing these issues is paramount for the successful transition of miRNA therapies into clinical practice, particularly in treating conditions like obesity and osteoporosis, where the precise modulation of gene expression is essential for achieving optimal therapeutic results. Consequently, overcoming these barriers—both in terms of delivery and specificity—must be prioritized to unlock the full potential of miRNA therapies in the management of obesity and osteoporosis, conditions that demand nuanced and targeted approaches for effective treatment.

## Conclusion

The exploration of miRNA therapies offers a revolutionary method for addressing the treatment of obesity and osteoporosis by targeting essential regulatory pathways involved in these multifaceted disorders [71]. The diverse and intricate roles that miRNAs play in modulating gene expression and cellular processes highlight their significant potential as therapeutic agents capable of impacting various biological functions [72]. As ongoing research progresses, it is critical to enhance our understanding of the precise mechanisms through which miRNAs exert their effects, particularly concerning adipogenesis—the process of fat cell formation—and osteogenesis, the formation of bone tissue [73].

The continued examination of various delivery mechanisms for miRNA-based therapies is paramount to improving their therapeutic efficacy and ensuring that these molecules are successfully directed to target tissues [67]. Recent advancements in nanotechnology, along with other sophisticated delivery systems, show great promise in addressing the challenges associated with the stability and bioavailability of miRNAs in clinical environments. Effective delivery systems can enhance the therapeutic potential of miRNAs by protecting them from degradation and facilitating their uptake by target cells [74].

Furthermore, robust clinical trials are essential to substantiate the therapeutic applications of specific miRNAs, as these studies will assess their safety and efficacy across diverse patient demographics [75]. Such rigorous evaluation is necessary not only to determine the effectiveness of miRNA therapies but also to elucidate their potential as biomarkers for monitoring disease progression. This understanding can subsequently aid in developing personalized treatment approaches tailored to the unique needs of individual patients, thereby enhancing overall therapeutic outcomes [76].

Additionally, emerging research investigating the influence of gut microbiota on miRNA activity presents an intriguing area for future studies. The gut microbiome plays a critical role in various metabolic processes, and understanding how it modulates miRNA expression could yield valuable insights into the relationship between metabolic health and bone integrity [77]. Insights gained from this research may inform more effective therapeutic strategies for managing obesity and osteoporosis, integrating the role of the microbiota in treatment plans.

In conclusion, although there are considerable challenges to overcome in applying miRNA research clinically, the potential benefits of miRNA therapies are substantial. By systematically addressing these challenges through dedicated research, the scientific community can develop innovative treatments that target obesity and osteoporosis while enhancing overall health outcomes for those affected. The future of miRNA-based therapies appears promising, positioning them as essential in managing these widespread health issues.

## Data Availability

The datasets gathered and examined in this study can be obtained from the corresponding author upon a reasonable request. Additionally, the names of the repositories and their reference numbers are accessible in online repositories.
